# A *Mycobacterium tuberculosis* Mbox controls a conserved, small upstream ORF via a translational expression platform and Rho-dependent termination of transcription

**DOI:** 10.1261/rna.080735.125

**Published:** 2026-02

**Authors:** Alexandre D'Halluin, Terry Kipkorir, Catherine Hubert, Declan Barker, Kristine B. Arnvig

**Affiliations:** Structural and Molecular Biology, University College London, London WC1E 6BT, United Kingdom

**Keywords:** *Mycobacterium tuberculosis*, PE–PPE, Rho, riboswitch, small protein

## Abstract

Magnesium is vital for bacterial survival, and its homeostasis is tightly regulated. Intracellular pathogens like *Mycobacterium tuberculosis* (*Mtb*) often face host-mediated magnesium limitation, which can be counteracted by upregulating the expression of Mg^2+^ transporters. This upregulation may be via Mg^2+^-sensing regulatory RNA such as the *Bacillus subtilis ykoK* Mbox riboswitch, which acts as a transcriptional “OFF-switch” under high Mg^2+^ conditions. *Mtb* encodes two Mbox elements with strong similarity to the ykoK Mbox. In the current study, we characterize the Mbox encoded upstream of the *Mtb pe20* operon, which is required for growth in low Mg^2+^/low pH. We show that this switch operates via a translational expression platform and Rho-dependent transcription termination, which is the first such case reported for an Mbox. Moreover, we show that the switch directly controls a small ORF encoded upstream of *pe20*. We have annotated this highly conserved uORF *rv1805A*, but its role remains unclear. Interestingly, a homologous gene exists outside the Mbox-regulated context, suggesting functional importance beyond magnesium stress. Overall, this study uncovers a dual mechanism of riboswitch-regulation in *Mtb*, combining translational control with Rho-mediated transcription termination. These findings expand our understanding of RNA-based gene regulation in mycobacteria, with implications for pathogenesis and stress adaptation.

## INTRODUCTION

Magnesium is required for a wide range of cellular functions in all domains of life and is the most abundant divalent cation in living cells (Smith et al. 1998). In bacteria, these functions include cell wall integrity, biofilm formation, macromolecular metabolism and function, making magnesium homeostasis essential (Thomas and Rice 2014; Subramani et al. 2016; Yamagami et al. 2021; Chatterjee et al. 2024). This represents an extra challenge for intracellular pathogens as host immune responses include mechanisms for restricting access to magnesium in certain cellular compartments such as the phagosome (Forbes and Gros 2001; Pokorzynski and Groisman 2023). To counteract these defense mechanisms, pathogens express an array of transporters to ensure adequate Mg^2+^ uptake. At least four types of magnesium channels and transporters regulate and maintain essential Mg^2+^ levels in prokaryotes: CorA, CorB/C, MgtA/B, and MgtE (Franken et al. 2022). *Mycobacterium tuberculosis* (*Mtb*) encodes CorA (Rv1239) and MgtE (Rv0362); however, *Mtb* does not encode homologs of MgtA/B, making the function of MgtC elusive (Alix and Blanc-Potard 2007).

Riboregulated, that is, RNA-based, stress responses are widespread in bacteria, with small RNAs and riboswitches being the most prominent elements. Riboswitches are located in the 5′ leader regions of mRNAs regulating gene expression in *cis*; they are composed of a highly structured ligand binding aptamer domain and an expression platform. The latter exerts gene expression control by either modulating premature termination of RNA polymerase (RNAP) and/or restricting access of the ribosome to the ribosome binding site (RBS) of the mRNA (Salvail and Breaker 2023). Nonpermissive control mechanisms may involve the formation of intrinsic terminators, unmasking of Rho-binding sites, or occlusion of Shine–Dalgarno (SD) sequence of the downstream open reading frame (ORF). The latter may in addition be associated with Rho-dependent termination of transcription within the ORF (Salvail and Breaker 2023). Binding of the specific ligand can either allow (“ON-switch”) or inhibit (“OFF-switch”) expression of the downstream gene (Breaker 2018; Schwenk and Arnvig 2018; Kavita and Breaker 2023). The genes regulated by riboswitches are often, but not always, involved in the metabolism or transport of the cognate ligand (Roth and Breaker 2009; Sherlock and Breaker 2020; Kavita and Breaker 2023). Riboswitch ligands range from sugars, amino acids, nucleotides, and cofactors to metal ions including Mg^2+^ (Barrick et al. 2004; Dann et al. 2007; Mccown et al. 2017; Breaker 2022). A magnesium-sensing riboswitch, referred to as Mbox, was first discovered in the *Bacillus subtili*s *ykoK* gene encoding a MgtE-type magnesium transporter (Townsend et al. 1995; Barrick et al. 2004; Ramesh and Winkler 2010). The Mbox is a transcriptional “OFF-switch”; magnesium binding to the aptamer leads to conformational changes of the RNA and the formation of an intrinsic terminator preventing *ykoK* expression. At low Mg^2+^ concentrations, the absence of the terminator is permissive to *ykoK* expression, which facilitates increased Mg^2+^ uptake (Ramesh and Winkler 2010).

Successful infection by *Mtb* requires its sensing of, and adaptation to, multiple microenvironments including different types of macrophages and their subcellular compartments such as phagosomes (Chandra et al. 2022; Samuels et al. 2022; Sholeye et al. 2022). *Mtb* has evolved mechanisms to either escape this organelle or to endure the hostile environment within by a range of adaptive responses (Ernst 2012; Ehrt et al. 2018; Huang et al. 2019). Riboswitches are likely to play a role in this adaptation by directly sensing host environments via specific metabolites. Several *Mtb* riboswitches have been predicted (Rfam RF00380) and their expression validated by RNA-seq, Term-seq, inline probing, and functional assays (Arnvig et al. 2011; Nawrocki et al. 2015; Kolbe et al. 2020; D'Halluin et al. 2023; Kipkorir et al. 2024a,b). These include two predicted Mbox aptamers upstream of Rv1535 and Rv1806 (*pe20 locus*), respectively. Both *loci* are upregulated in low magnesium (Walters et al. 2006), and in a recent study Kolbe et al. demonstrated that magnesium-dependent control resides within the leader, not the promoter of *pe20* (Kolbe et al. 2020). Moreover, the two *Mtb* aptamers show a high level of structural similarity to the *B. subtilis ykoK* aptamer, although some results suggest that these interact differently with divalent cations including Mg^2+^ (Bahoua et al. 2021). The *pe20 locus* (encoding PE20, PPE31, PPE32, PPE33, Rv1810, and MgtC) is associated with magnesium homeostasis and acid stress, and PE20–PPE31 have been shown to be necessary for maintaining growth in a combination of low Mg^2+^ and low pH, conditions that mimic the phagosomal environment (Walters et al. 2006; Wang et al. 2020). The function of Rv1535 remains unknown.

We recently mapped premature termination of transcription in *Mtb* at genome-scale and identified hundreds of RNA leaders with an abundance of potential new riboswitches and translated small upstream ORFs (uORFs) (D'Halluin et al. 2023). We validated predicted riboswitches and demonstrated that both *Mtb* Mbox leaders were associated with premature, Rho-dependent termination of transcription upstream of the annotated ORFs (D'Halluin et al. 2023).

Here, we show that *pe*–*ppe*-associated Mboxes are widely conserved across *Mycobacterium* and are cotranscribed with *mgtC* in *Mtb*. The *Mtb* Mbox upstream of *pe20* is unusual as it combines a translational expression platform with a Rho-dependent transcription terminator. This is to our knowledge the first translational Mbox to be described. Using translational reporter fusion constructs, we show that a conserved uORF located between the Mbox and *pe20* is highly expressed. This peptide is highly conserved in the context of Mboxes across the *Mycobacterium* genus. While its function remains opaque, a paralog of this ORF is expressed from an additional, Mbox-independent *locus* in *Mtb*, supporting the biological and regulatory importance of this peptide.

## RESULTS

### Conservation and genomic context of *M. tuberculosis* Mboxes

**FIGURE 1.**
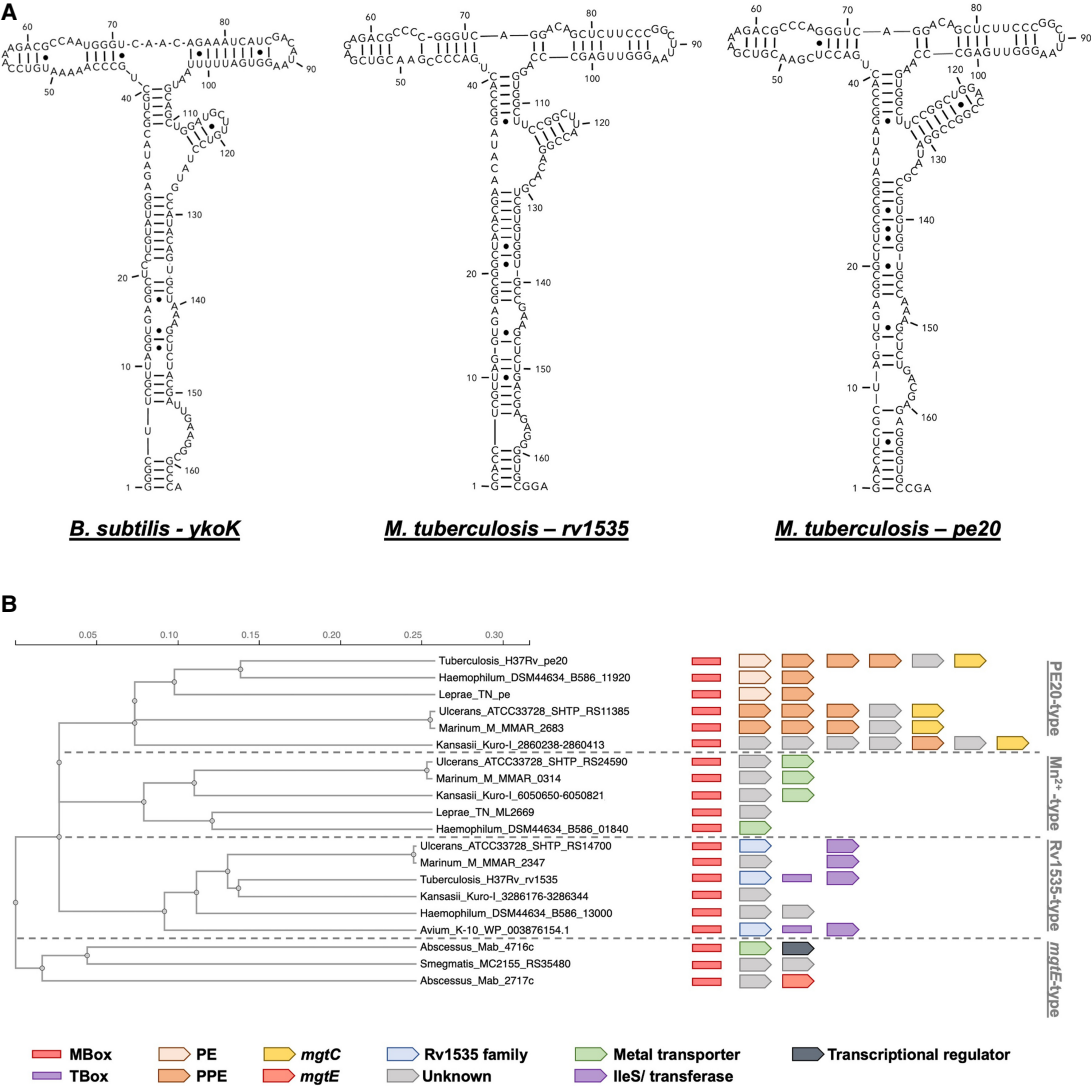
Conservation of Mbox elements. (*A*) Mbox aptamer structures from *B. subtilis ykoK* and the two *Mtb* aptamers from *rv1535* and *pe20* (*rv1806*). Structures were predicted using RNAstructure web server (Reuter and Mathews 2010) and drawn by extracting the bracket-dot plot to RNAcanvas (Johnson and Simon 2023). (*B*) Distribution of Mboxes and their associated genes in *Mycobacteria*. These can be split into the four types indicated, based on the aptamer and their downstream sequences. Notably, the two *Mtb* elements fall into different groups.

Two Mbox aptamers have been identified within the *Mtb* H37Rv genome (Rfam RF00380). We used these sequences to predict their structures and compare these to the Mbox consensus structure from Rfam. The results, shown in Figure 1A, indicate a high degree of similarity between the two *Mtb* aptamers and the *B. subtilis ykoK* element, suggesting these are functional Mg^2+^-sensing elements as reported in the case of *rv1535* by (Bahoua et al. 2021).

Next, we investigated the conservation of the element and its context across the *Mycobacterium* genus. Both elements have been shown to be associated with multiple uORFs, and at least one of these is translated (u2, D'Halluin et al. 2023). Based on a phylogenetic analysis of the two *Mtb* elements and Mboxes from other species, we identified four classes of mycobacterial Mboxes, represented by *pe20*-type, manganese-type (Mn^2+^-type), *rv1535*-type, and *mgtE*-type elements, respectively; these classes are further supported by a conserved gene synteny (Fig. 1B).

The *mgtE*-type is the only Mbox found in the nonpathogenic *Mycobacterium smegmatis*, and its genomic neighborhood shows that the genes immediately downstream encode predicted metal transporters (MTs) and/or associated proteins (e.g., MgtE), or proteins of unknown function. The other three branches are seen across fast- and slow-growing pathogenic mycobacteria.

The first branch, the *pe20*-type, is almost exclusively found upstream of multiple *pe*–*ppe* genes, which in *Mtb*, *Mycobacterium ulcerans*, *Mycobacterium marinum*, and *Mycobacterium kansasii* are followed by genes of unknown function (GUF) and MT (as MgtC was originally annotated as a magnesium transporter). The second Mn^2+^-type branch includes a cluster of manganese transporter-type downstream from the riboswitch, a constellation that is not seen in *Mtb*. The third *rv1535*-type branch is found upstream of GUF like *rv1535* but followed by a cluster of T-box/*ileS* elements or various transferases. These results suggest the regulation of metal transporters by both Mboxes is well-conserved across mycobacteria, while the *pe*–*ppe* clusters and mosaic appearance suggest insertion events may have taken place in pathogenic/slow-growing species.

### Rho-dependent premature termination of transcription within Mbox leaders

TSS mapping and RNA-seq suggest that the *rv1535* mRNA is monocistronic, while the *pe20* mRNA is polycistronic spanning *pe20* to *mgtC* (Supplemental Fig. 1; Arnvig et al. 2011; Cortes et al. 2013; D'Halluin et al. 2023). Importantly, the entire polycistronic *pe20* operon is upregulated during growth in low magnesium controlled by the Mbox (Walters et al. 2006).

**FIGURE 2.**
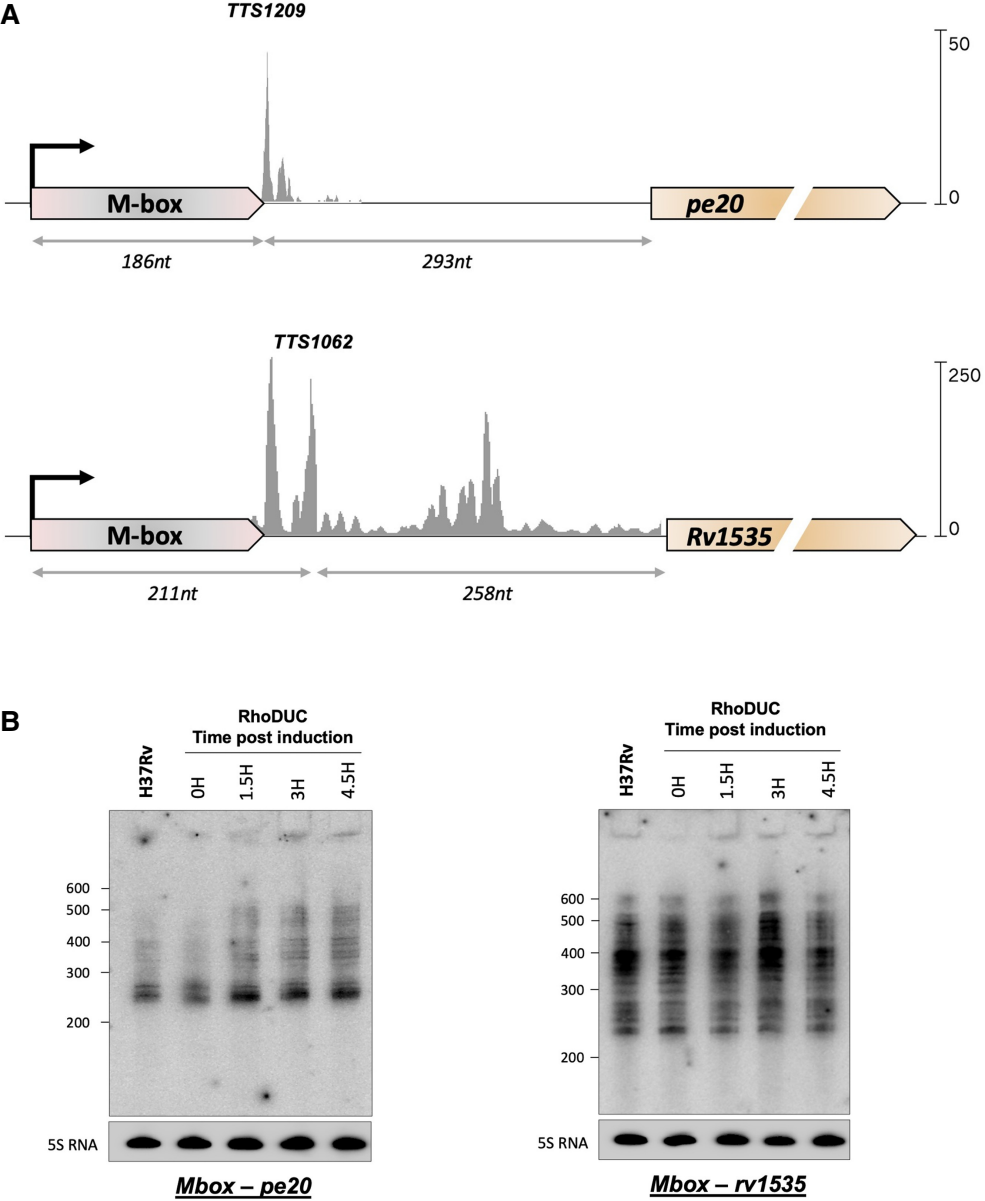
Premature termination of transcription within *Mtb* Mbox *loci*. (*A*) The two Mbox-associated genes, *rv1535* and *pe20*, are shown with their respective leaders: TSS from Cortes et al. (2013), Term-seq data and Transcription termination sites (TTS) from D'Halluin et al. (2023). Distances from TSS to dominant TTS peaks and further to the start codons are indicated. (*B*) Northern blot with log-phase total RNA from *Mtb* H37Rv and from RhoDUC (Botella et al. 2017) following depletion of Rho. Total RNA was separated on an 8% acrylamide gel, electroblotted, and probed for leader sequences distinct for the two genes, ∼180 nt downstream from the TSS. The 5S RNA was probed as a loading control.

We recently mapped premature transcription termination (TTS) in *Mtb* genome-wide and identified two dominant TTS associated with the Mbox leaders (D'Halluin et al. 2023). TTS1062 is located ∼210 nt downstream from the *rv1535* TSS and 40 nt downstream from the aptamer. TTS1209 is located ∼185 nt downstream from the *pe20* TSS and 10 nt downstream from the aptamer (Fig. 2A).

In both *loci*, multiple smaller peaks are flanking the TTS, suggesting a degree of flexibility in the TTS. Both TTSs were located a significant distance (>200 nt) upstream of their annotated ORFs, revealing the premature termination of transcription within the two leader regions, and neither was associated with canonical intrinsic terminator structures.

In our *Mtb* TTS mapping, we predicted and validated Rho-dependent termination using RhoTermPredict (Di Salvo et al. 2019) and depletion of Rho using the Rho-DUC strain (Botella et al. 2017; D'Halluin et al. 2023). Two Rho-binding (*rut*) sites were predicted in each Mbox leader: one in each aptamer (T5468 and T6425) and one between aptamers and annotated ORFs (T5469 and T6426), while the mapped TTS1209 and TTS1062 are located between these (Table 1; Fig. 2A). The calculated readthrough (RT) scores for the mapped TTS after anhydrous tetracyclin (ATc) induced depletion of Rho validated that transcription termination was in fact due to Rho (D'Halluin et al. 2023). To further confirm Rho-dependent, premature termination of transcription, we performed northern blotting on RNA from H37Rv and from Rho-depleted cultures probing for both Mboxes.

**TABLE 1.** Mapped transcription termination sites (TTS), predicted Rho-dependent terminators (RTPs) and their locations according to D'Halluin et al. (2023)

Mbox gene	Mapped TTS	RTP number^a^	*rut* site start	*rut* site end	Distance from TSS^b^	Distance to annotated ORF^b^
*rv1535*	1735718	T5468	1735526	1735604	17	−372
		T5469	1735816	1735894	307	−82
*pe20*	2047779	T6425	2047633	2047711	38	−361
		T6426	2047887	2047965	292	−107

^a^Terminator number according to RhoTermPredict (see text for details).

^b^Nucleotide distance between transcription start site (TSS) and mapped TTS; positive values indicate TTS is downstream, negative values that TTS is upstream.

The homology between the Mbox aptamers from *rv1535* and *pe20* made it impossible to design a 5′ probe that could distinguish between the two transcripts. To ensure that the signals were specific for either *pe20* or *rv1535*, we used a probe that was located 180 nt into the transcripts beyond the homologous regions (Supplemental Fig. 2), and as a result, transcripts shorter than this could not be detected.

Several strong signals between 200 and 300 nt roughly corresponded to the TTS mapping, suggesting multiple points of premature termination of transcription within both leaders (Fig. 2B). In H37Rv and in Rho-DUC time 0, we observed limited readthrough beyond 300 nt for *pe20*, while *rv1535* displayed multiple larger signals primarily ∼400 nt consistent with the TTS pattern. Depletion of Rho led to an increase in larger transcripts for *pe20* suggesting increased readthrough, that is, reduced termination. In contrast, the *rv1535* termination pattern changed only marginally over the Rho depletion time course, suggesting that Rho plays a greater role for *pe20* regulation as compared to *rv1535*.

### The regions downstream from the Mbox aptamers harbor multiple uORFs

Ribosome profiling demonstrates that the regions between the Mbox aptamers and the two annotated open reading frames (ORF)s (*rv1535* and *pe20*, respectively) are bound by ribosomes in agreement with ongoing translation upstream of the annotated genes (Sawyer and Cortes 2022; Smith et al. 2022; D'Halluin et al. 2023). Sequence alignment of *rv1535* and *pe20* leaders with other *pe20*-type leaders indicated several regions of conservation including near-identical SD sequences located at the end of the aptamer (SD1) and a second, highly conserved SD (SD2) further downstream. The ORF downstream from SD1 (upstream ORF1/uORF1) shows poor conservation, while the uORF downstream from SD2 (uORF2) is highly conserved (Supplemental Fig. 2). Moreover, we have previously shown that uORF2 from both loci is expressed (D'Halluin et al. 2023).

**FIGURE 3.**
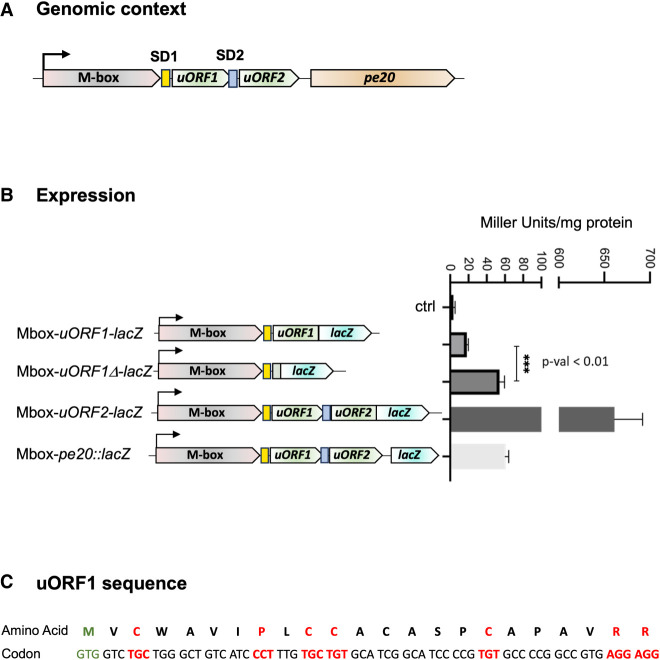
Expression of *pe20* uORFs. To ascertain expression of uORF1 and uORF2 from the *pe20* operon, we made translational *lacZ*-fusions and measured β-galactosidase (β-gal) activity of the different constructs. Experiments were done in triplicate, and differences in expression were tested with a *t*-test (*P* < 0.01). (*A*) Genomic context of *pe20* and the uORFs associated (green) including SD1 (yellow box) and SD2 (blue box). (*B*) Schematic showing each reporter construct (*left*) and their expression in Miller units (*right*). uORF1D refers to a truncated version of uORF1 encoding only its first two codons. (*C*) uORF1 sequence with amino acids and their codons. Start codon is shown in green, and rare codons (<5/1000 frequency) are shown in red.

To characterize the relationship between the *pe20* Mbox and the two uORFs, we first investigated expression using translational *lacZ*-fusions. All constructs included the 5′ leader from the TSS and were gradually extended downstream to the end of uORF1 (Mbox-*uORF1-lacZ*), the end of uORF2 (Mbox-*uORF2-lacZ*), or the start codon of *pe20* ORF (Mbox-*pe20::lacZ*), respectively. All were fused in-frame to *lacZ*, expressed from a heterologous, constitutive promoter and integrated into the *M. smegmatis* genome in single copy (Fig. 3). Next, we performed β-galactosidase (β-gal) assays, which showed that Mbox-*uORF2-lacZ* expression was >10-fold higher than Mbox-*pe20::lacZ* (∼650 Miller units compared to 60 Miller units), while Mbox-*uORF1-lacZ* was only slightly higher than the background (Fig. 3B). To validate the start codons of the two uORFs, we mutated each to non-start codons (GTG to GTC and ATG to ACG for uORF1 and uORF2, respectively). This reduced β-gal activity significantly in both constructs, suggesting reduced expression in support of the annotated translation start sites, although Mbox-*uORF2-lacZ* expression was still higher than Mbox-*pe20::lacZ* expression (Supplemental Fig. 3).

As the translation initiation regions (TIR) for uORF1 and uORF2 (i.e., SD1 and SD2 and their distances to the start codons) were almost identical, and we had not observed any premature TTS in the region, we reasoned that the coding region of uORF1 was responsible for the lower β-gal activity. To explore this possibility, we deleted the majority of uORF1 from the Mbox-*uORF1-lacZ* construct except the first two codons (Mbox-*uORF1*Δ*-lacZ*) and measured *lacZ* expression.

The results, shown in Figure 3B, indicate that expression of this truncated uORF1 was 2.5-fold higher than that of the full-length uORF1, suggesting that the uORF1 coding region did indeed suppress β-gal activity. As uORF1 contains several rare (≤5/1000) codons, that is, TGC, CCT, TGC, TGT, TGT, AGG, AGG (Fig. 3C), we assume that this was due to poor translation elongation, but alternative explanations such as the uORF1 peptide interfering with β-gal activity cannot be ruled out at this stage.

### The *pe20* Mbox operates via a translational expression platform

In conjunction, the Rho-dependent premature termination of transcription, the highly conserved SDs at the end of the aptamer, located upstream of a well-expressed conserved uORF, made us speculate that the *pe20* Mbox operates via a translational expression platform.

A functional translational expression platform requires the potential for the SD to be masked, for example, by a pyrimidine-rich region (an αSD) that in turn can be sequestered by an ααSD under different conditions.

**FIGURE 4.**
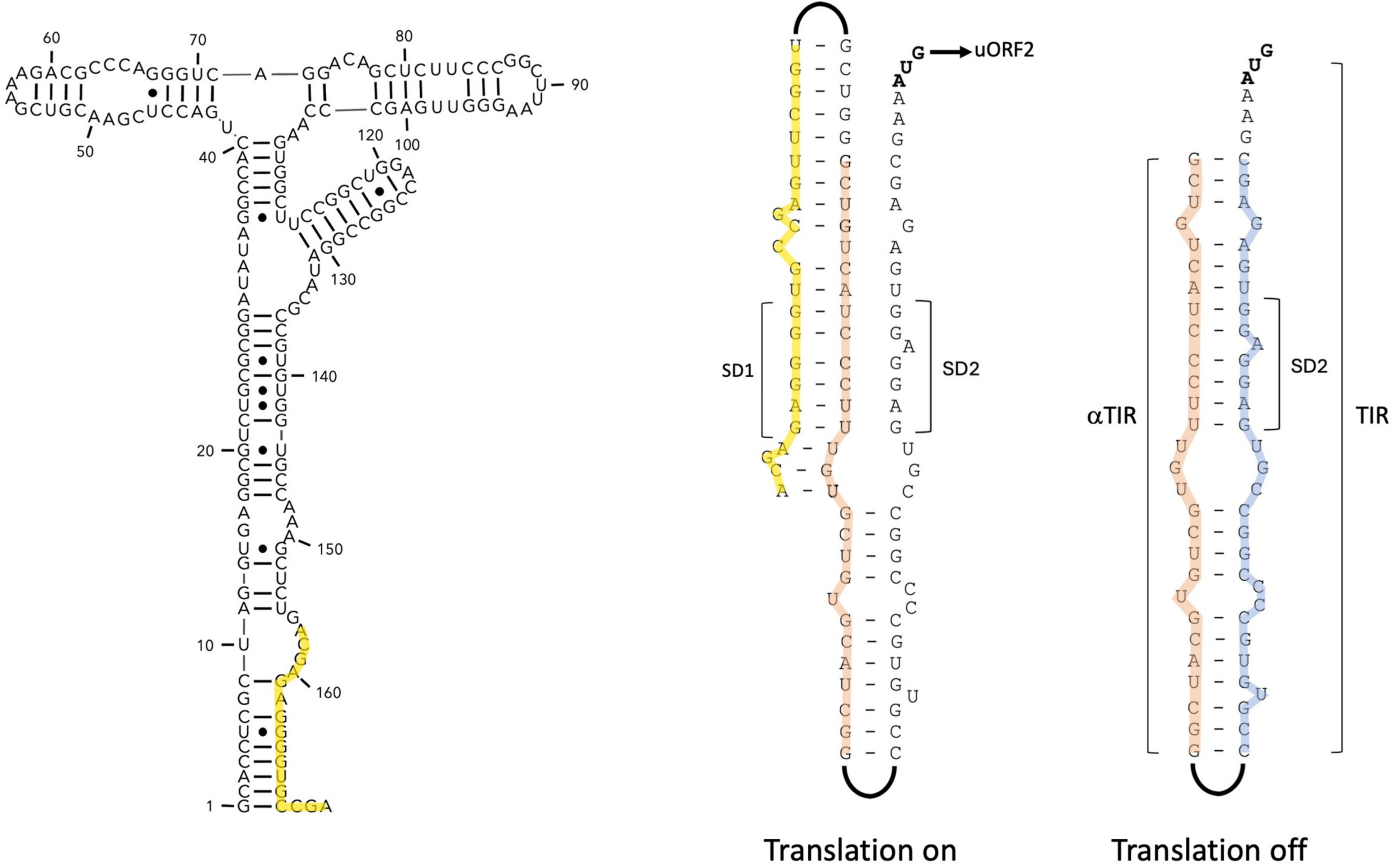
Model for a translational expression platform. The figure shows how the translation initiation region (TIR, blue) can be sequestered by base-pairing with the αTIR (orange), which in turn can base-pair with the ααTIR (yellow), depending on the conformation of the aptamer. Structure of the aptamer is shown on the *left* with part of the ααTIR shown in yellow.

We identified such a region approximately halfway between SD1 and SD2. This αSD and its flanking regions have the potential to pair with the entire translation initiation region (TIR) of uORF2 (shown in blue in Fig. 4) or alternatively, with the aptamer-associated SD1 and its flanks (yellow in Fig. 4).

To explore this hypothesis further, we measured uORF2 expression after introducing mutations that could interfere with the proposed interactions. One was the abolishing of the uORF1 start codon, the rationale being that this would partially unmask SD1, thereby favoring the SD1–αSD interaction, leading to an increase in *uORF2-lacZ* expression.

**FIGURE 5.**
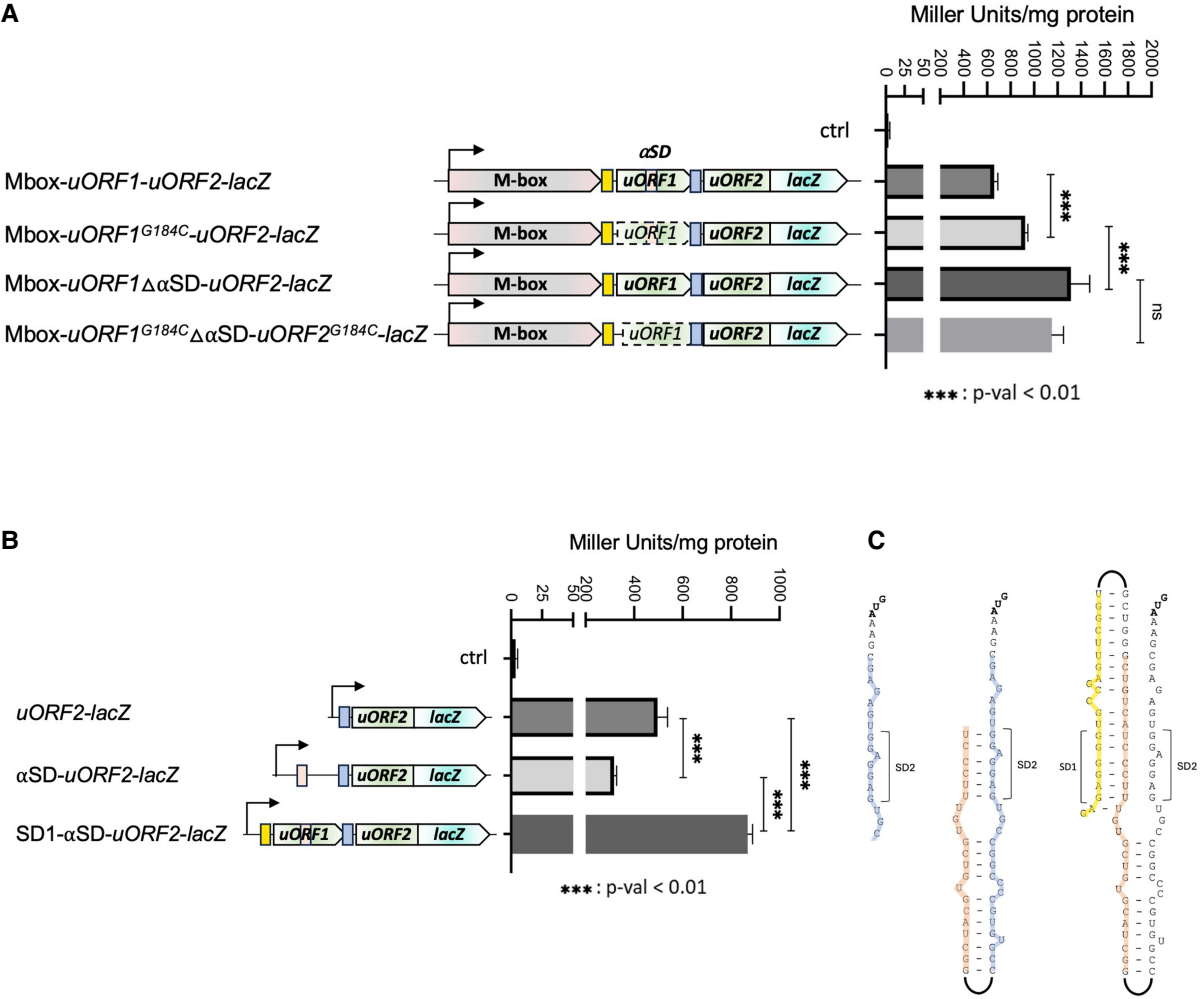
Testing the model for a translational expression platform. (*A*) Reporter constructs assessing the effect of uORF1 changes on uORF2 expression; changing the start codon of uORF1 to a no-start (G184C), deleting the proposed αSD, which is part of uORF1 or a combination of the two. (*B*) Effect of gradual extension of the region upstream of uORF2. Expression decreases when αSD is included and increases again when SD1 (ααSD) is included. (*C*) Structures indicating how the reporter constructs relate to the model proposed in Figure 4. Experiments were done in triplicate, and differences in expression were tested with a *t*-test (*P* < 0.01).

Similarly, deleting the αSD should also lead to higher expression of uORF2, as SD2 would no longer be sequestered. The results, shown in Figure 5A, indicate a moderate (∼1.3-fold) but significant increase in expression, when the start codon of uORF1 was changed (Mbox-*uORF2^G184C^-lacZ*), and a larger (approximately twofold) increase in expression, when the αSD was deleted (Mbox-ΔαSD*-uORF2-lacZ*). Combining the two mutations did not result in an additive effect, suggesting they involved the same mechanism (Fig. 5A). These results support a model in which SD1 (ααSD), αSD, and SD2 interact to control the expression of uORF2-*lacZ*.

To further validate this model, we assessed the contribution of each element by gradually extending the region between uORF2 and the Mbox in uORF2-*lacZ* fusions (Fig. 5B). The SD2-uORF2 construct displayed β-gal expression levels of ∼500 Miller units, and the addition of the αSD motif reduced the β-gal expression by ∼35%. However, a further extension including the ααSD motif led to a substantial increase in uORF2 expression. This is likely due to the unmasking of SD2 and corroborates our model of a translational expression platform controlling uORF2 expression.

Our results support a model in which uORF2 is controlled by a translational Mbox riboswitch combined with Rho-dependent termination of transcription. Based on sequence homology, we propose that the *rv1535* Mbox likewise operates via a translational expression platform. To the best of our knowledge, these are the first examples of an Mbox translational expression platform and Rho-dependent termination of transcription.

### An Mbox-independent homolog of uORF2 encoded in a separate *Mtb locus*

**FIGURE 6.**
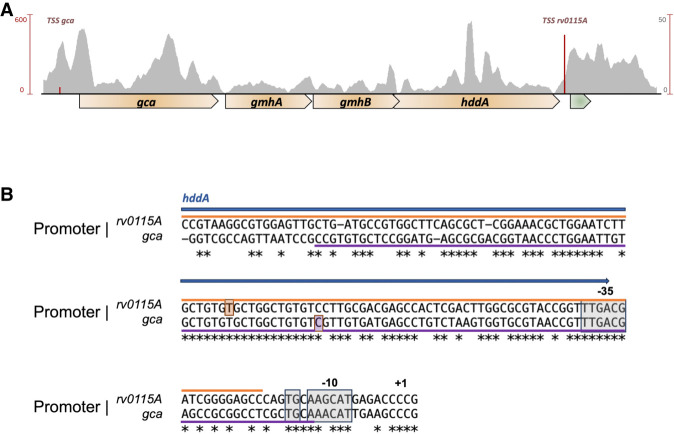
The Rv0115A locus. BLAST identified Rv0115A to be a homolog of Rv1805A. (*A*) Rv0115A (green) is encoded downstream from the *gca* operon (golden) but transcribed from its own promoter. (*B*) Alignment of the promoter regions of *gca* and *rv0115A* shows a very high degree of similarity, suggesting a duplication event. The blue arrow indicates *hddA* coding sequence upstream of *rv0115A*. The promoter elements, −35, extended −10, and −10, are highlighted in gray. PhoP binding regions, according to TBDB, are shown in orange and purple with their respective centers boxed in the same color.

Considering the high conservation between uORF2 in the *rv1535* and *pe20 loci*, we carried out deeper sequence searches and identified a third homolog of the uORF2 region including its SD downstream from the *gca*-*gmhA*-*gmhB*-*hddA* operon. This *locus* has been acquired by horizontal gene transfer (Becq et al. 2007), and the uORF2 homolog is annotated as Rv0115A (Fig. 6A).

We identified two TSS and associated promoter motifs within this *locus*. The first drives the transcription of the *gca-hddA* operon, which terminates downstream from *hddA* (D'Halluin et al. 2023). The second drives the transcription of *rv0115A*, and potentially also a second ORF, *rv0115B*. The *gca* and *rv0115A* promoters have similar unusual motifs in the form of an AANCAT −10 hexamer, an extended −10 motif (TGN), a perfect −35 hexamer, and in the case of *cga*, a Cytidine TSS (Fig. 6B).

A further alignment of the promoter regions from −120 to a few base pairs downstream from the mapped TSS, had a remarkable similarity more than 100 bp upstream of the TSS suggestive of a gene duplication event (Fig. 6B). There are no Mbox elements associated with the leaders of these genes, but according to TBDB (http://tbdb.bu.edu/tbdb_sysbio/MultiHome.html), both promoters include a binding site for PhoP, linking expression to pH stress (Abramovitch et al. 2011).

**FIGURE 7.**
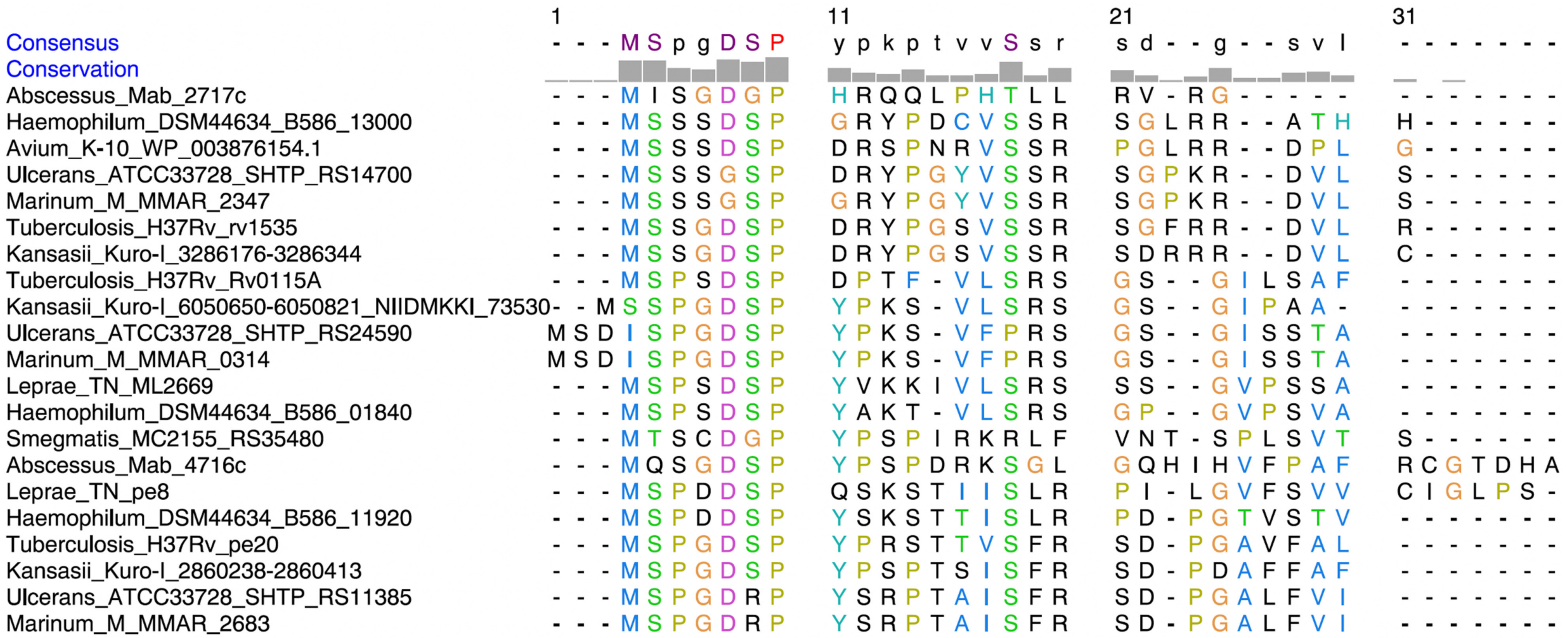
Conservation of uORF2 within mycobacteria. Alignment of Mbox-associated uORF2 extracted from Figure 1B and *Mtb* Rv0115A peptides showed high conservation of several residues mainly at the N-terminal sequence, including 100% conservation of a proline at position 7 in most peptides. Consensus sequence and amino acid conservation were assessed using Chimera (Meng et al. 2006).

Alignment of uORF2 peptide homologs including Rv0115A across mycobacterial species reveals a well-conserved N-terminal region, including a universally conserved proline residue (Fig. 7). This peptide is specific for mycobacteria, which indicates that uORF2 peptides and their homologs have functions uniquely associated with this genus. Based on this finding, we suggest renaming uORF2 from the *pe20* operon Rv1805A.

### No evident role for Rv1805A in biofilm formation during magnesium stress

Realizing the ubiquitous presence of Rv1805A homologs, we sought to find a role for this peptide. PE20 and PPE31 are necessary for *Mtb* growth in conditions of low Mg^2+^ combined with low pH (Wang et al. 2020). To probe a potential role of uORF2 in this process, we exploited the fact that magnesium is required for biofilm formation in mycobacteria (Chatterjee et al. 2024) and leveraged the trick that *M. smegmatis*, a closely related species*,* has no homolog of *pe20* locus.

**FIGURE 8.**
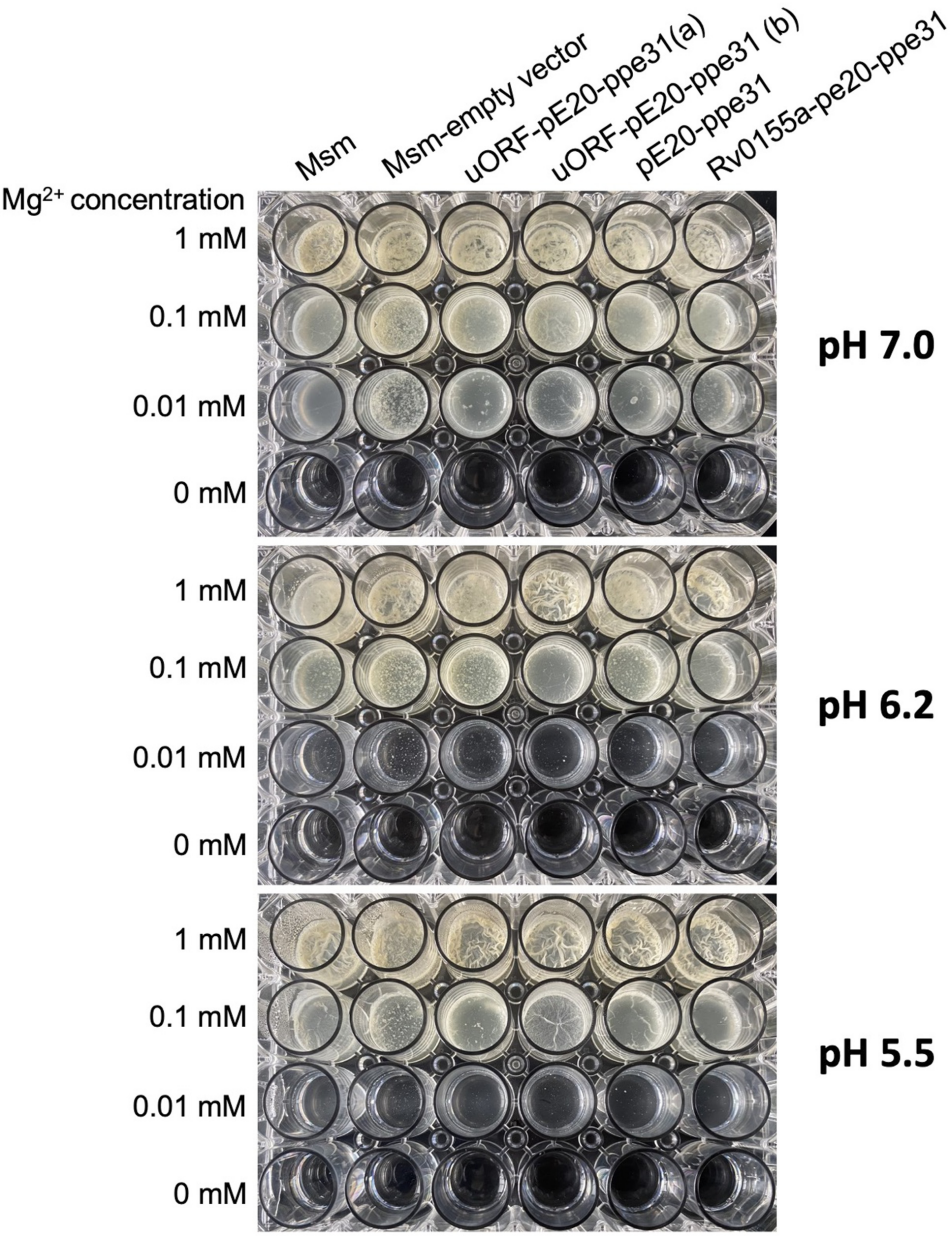
Biofilm formation in *M. smegmatis* during Mg^2+^-depletion and acid stress. Cultures of *M. smegmatis* were grown to mid-log phase, washed in Mg^2+^-free medium, and resuspended in 1 mL of the indicated medium at OD 0.01. Plates were sealed in plastic bags and left for static incubation at 37°C for a week. Plates shown are representative of three independent experiments.

In agreement with the literature, the growth and biofilm formation of *M. smegmatis* were compromised in low Mg^2+^, and this phenotype was exacerbated at acidic pH values (Fig. 8). We tested whether the expression of *pe20–ppe31* or *rv1805A–pe20–ppe31* might rescue this phenotype by transforming *M. smegmatis* with plasmids expressing the cognate genes. The results in Figure 8 indicate no visible difference between strains expressing *pe20–ppe31* with or without *rv1805A* or *rv0115A*; further investigations are required to identify a role of this peptide and its homologs in mycobacterial biology.

## DISCUSSION

In the current study, we have revealed a novel complex riboregulatory system, which controls *pe20* gene expression in *Mtb*. Our results show that premature termination occurring in the 5′ leader of *pe20* (and *rv1535*) relies on Rho-dependent termination of transcription (D'Halluin et al. 2023). Moreover, the *pe20* Mbox contributes a translational expression platform, where the translation initiation region including the SD of the first gene in the operon can be sequestered by an αSD motif. This is also, to the best of our knowledge, the first example of a translationally controlled Mbox. This type of control is consistent with the scarcity of intrinsic terminators in *Mtb*, and it echoes the finding that a mycobacterial T-box is the only known T-box with a translational expression platform (Sherwood et al. 2018) Finally, we identified a highly conserved uORF (*rv1805A*), which is the primary regulated ORF within the *pe20* operon and, based on homology, likely also in the *rv1535* operon.

Expression of the *pe20* operon is suppressed via its leader by high Mg^2+^ concentrations similar to the Mbox-controlled *ykoK* gene in *B. subtilis* (Walters et al. 2006; Ramesh and Winkler 2010; Kolbe et al. 2020). *pe20* and *ppe31* are critical for magnesium uptake in low-pH/low-magnesium conditions suggesting that the gene products form (part of) a magnesium transporter (Wang et al. 2020; Feng et al. 2021). We propose that the Mbox–*rv1805A* module acts as the key regulatory gate, enabling expression of the magnesium-responsive PE/PPE transporter complex only under specific environmental conditions, such as low Mg^2+^ and acidic pH.

The structure of the *pe20* operon, including the presence of *mgtC*, raises questions about its ancestry. Given what is known about *pe–ppe* gene expansion (Fishbein et al. 2015) and what we have observed in other riboswitch-controlled *pe/ppe loci,* i.e., the *Cbl-ppe2-cobQ locus*, and the PE-containing uORF recently identified downstream from the *Mtb* glycine riboswitch (D'Halluin et al. 2023; Kipkorir et al. 2024a), it is tempting to speculate that an early *pe*(*–ppe*) element invaded the current *pe20 locus* and subsequently expanded whereby Rv1805A became the first gene in this operon.

A recent study suggests that the *rv1535* Mbox, and by extension likely also the *pe20* Mbox, associates with other divalent cations in addition to Mg^2+^ (Bahoua et al. 2021), while Kolbe et al. have demonstrated strong Mg^2+^-dependent control of *pe20* expression via its leader (Kolbe et al. 2020). Regardless of the identity of the cognate ligand, our results suggest an ability to alternate between two structures: a nonpermissive (ligand-bound) structure that sequesters SD1, allowing αSD/αTIR to pair with SD2/TIR thereby preventing translation of uORF2/Rv1805A. This could in turn lead Rho-dependent termination of transcription, which will affect the entire operon (Hao et al. 2021; Molodtsov et al. 2023). We note, however, that according to Term-seq results, the primary TTS is located upstream of *rv1805A*, suggesting that Rho-dependent termination does not depend on translation of this ORF. An alternative explanation of our results could therefore be that the pyrimidine-rich region that we have annotated as αSD, might act as a Rho-binding (*rut*) site that would be masked by translation of uORF1. Deleting this region increased expression 2.5-fold, likely due to reduced termination of transcription or by unmasking of SD2 or both. The marginal increase in the expression of uORF2 in the context of an untranslated uORF1 (Mbox-*rv1805A^G184C^-lacZ*, Fig. 5) and the conservation of the ααSD–αSD interaction, suggests a functional interaction. The two models are not mutually exclusive, and further experiments will elucidate the structural and mechanistic basis underlying the regulation. Along the same lines, we note that expression of uORF1 might affect the activity of LacZ, although this is unlikely to affect the overall conclusions.

What is the function of Rv1805A and its homologs? Given its conservation and position upstream of *pe20*, we hypothesize that Rv1805A may act as a regulatory peptide modulating the activity or assembly of the PE20–PPE31 complex. Alternatively, it may serve as a structural component of a magnesium-responsive transporter. Conservation between Rv1805A, the Rv1535 uORF2 (Rv1535A), and Rv0115A, and their associations with magnesium and pH stress, suggests important roles for these peptides during infection. Future work will focus on identifying interaction partners of Rv1805A and assessing its role in magnesium uptake and stress responses.

In conclusion, our findings reveal a previously unrecognized mode of riboswitch control in *Mtb*, where a translational Mbox integrates with Rho-dependent termination to regulate a conserved uORF. This multilayered modus operandi underscores the sophistication of RNA-based regulation in *Mtb* stress adaptation.

## MATERIALS AND METHODS

### Strains and cultures

Strains used in this study are listed in Supplemental Table 1. *Mtb* H37Rv and *M. smegmatis* MC^2^ 155 were cultured on solid media Middlebrook agar 7H11 supplemented with 10% OADC (Sigma-Aldrich), 0.5% glycerol, and 50 µg/mL hygromycin if appropriate. Liquid cultures were done in Middlebrook 7H9 supplemented with 10% ADC (Sigma-Aldrich), 0.5% glycerol, 0.05% Tween 80, and 50 µg/mL hygromycin where appropriate. Cultures were harvested at an OD_600 nm_ ∼0.6 for mid-log phase.

*Mtb* RhoDUC strain, a gift obtained from Professor Dirk Schnappinger, was grown as previously described with 50 µg/mL hygromycin, 20 µg/mL kanamycin, and 50 µg/mL zeocin (Botella et al. 2017; D'Halluin et al. 2023). When the cultures reached an OD_600 nm_∼0.6, depletion of Rho was induced using 500 ng/mL of anhydrotetracycline. Cells were harvested after 0, 1.5, 3, and 4.5 h.

*Escherichia coli* DH5α was used for cloning the *lacZ* fusion reporters and was cultured on solid LB 1.5% agar supplemented with 50 µM of 5-bromo-4-chloro-3-indolyl-β-d-galactopyranoside (X-gal) or in liquid LB supplemented with 250 µg/mL hygromycin.

### Plasmid constructions and primers

Plasmids and primers used in this study are listed in Supplemental Tables 1 and 2. pIRATE plasmids, described in D'Halluin et al. (2023), were used for *lacZ* translational fusion reporters and for Beta-galactosidase assay. Reporters were constructed using Gibson assemblies with oligos (Sigma-Aldrich) or geneBlocks (IDT) listed in Table 3 between HindIII and NcoI sites. Point mutations and deletions were generated using the Q5 Site-Directed Mutagenesis Kit (New England Biolabs). Plasmids were cloned in *E. coli* DH5α, extracted and sequenced by Sanger sequencing. Plasmids were transformed into *M. smegmatis* by electroporation and selected on Middlebrook 7H11 agar plates containing 50 µg/mL hygromycin.

### RNA extraction and northern blotting

*Mtb* H37Rv were stopped using 37.5% of cold ice and centrifuged for 10 min at 5000 rpm at 4°C. Total RNA was extracted as previously described using the FastRNA Pro Blue kit (MP Biomedicals) according to the manufacturer's protocol (Arnvig et al. 2011; D'Halluin et al. 2023). RNA concentration and purity was assessed using the NanoDrop 2000 (Thermo Fisher), residual genomic DNA was removed using TURBO DNase (Thermo Fisher), and RNA integrity was assessed with 2100 Bioanalyzer (Agilent). Ten micrograms of total RNA was separated on a denaturing 8% acrylamide:bis-acrylamide (19:1) gel and transferred to a nylon membrane. An RNA probe was synthetized using the mirVana miRNA Probe Synthesis Kit (Ambion) to reveal the *pe20* and *rv1535* Mbox transcripts and labeled with 3 µM final concentration of ^32^P α-UTP (3000Ci/mmol; HARTMANN Analytic GmbH). Northern blots were revealed using radiosensitive screens and visualized on a Typhoon FLA 9500 phosphoimager (GE Healthcare).

### Beta-galactosidase activity

*M. smegmatis* carrying the *lacZ* reporter fusions were cultured at OD_600 nm_ ∼0.6 and centrifuged for 10 min at 5000 rpm. Pellets were washed four times in Z-buffer composed of 60 mM Na_2_HPO_4_, 40 mM NaH_2_PO_4_, 10 mM KCl, 1 mM MgSO_4_ and lysed using beads with the FastPrep Biopulverizer (MP Biomedicals). The supernatant was kept after centrifugation, and the protein level was assessed using a Bradford yield with the BCA Kit (Thermo Fisher) following the manufacturer's recommendations. Beta-galactosidase were done using the beta-Galactosidase Assay Kit (Thermo Fisher) following the manufacturer's protocol. Proteins were preincubated for 5 min at 28°C before addition of ONPG.

### Biofilm formation

*M. smegmatis* expressing *pe20–ppe31, rv1805A–pe20–ppe31*, or *rv0115A + pe20–ppe31* was grown to mid-log phase, washed in Mg^2+^-free medium, resuspended in 1 mL of the indicated medium at OD 0.01, and seeded in 24 well plates. Plates were sealed in plastic bags and left for static incubation at 37°C for a week. Biofilm formation was monitored every day for a week.

### Folding, sequence conservation, and distribution across mycobacteria

Representative genomes of several mycobacteria were selected for sequence conservation: *Mycobacterium tuberculosis* H37Rv (NB_000962), *Mycobacterium leprae* TN (AL450380), *Mycobacterium avium* K10 (NZ_CP106873), *M. kansasii* Kuro I (AP023343), *M. ulcerans* ATCC33728 (NZ_AP017624), *M. marinum* M (CP000854), *Mycobacterium abscessus* ATCC19977 (NC_010397), *Mycobacterium haemophilum* DSM 44634 (CP011883), and *M. smegmatis* MC^2^155 (NZ_CP009494).

The aptamer sequences of the Mboxes were extracted from RFam database (Rfam RF00380) (Nawrocki et al. 2015) and extended to the next annotated ORF. DNA and peptidic sequences were aligned using ClustalW (Thompson et al. 1994), and alignment was strengthened using T-coffee (Notredame et al. 2000). The conservation of uORF2 across mycobacteria was determined using Blast (Altschul et al. 1990), and amino acid sequences were aligned using ClustalW (Thompson et al. 1994) and Chimera (Meng et al. 2006). The phylogenetic tree was generated by Clustal Omega using the sequences from the aptamer sequence to the start codon of the next in-frame annotated ORF (Sievers et al. 2011). Aptamer secondary structures were predicted using the RNAstructure web server for RNA secondary structure prediction (Reuter and Mathews 2010). The resulting connectivity table (CT) file was then uploaded to RNAcanvas (Johnson and Simon 2023) for visualization and structural editing

## SUPPLEMENTAL MATERIAL

Supplemental material is available for this article.

## Figures and Tables

**Alexandre D‘Halluin:**
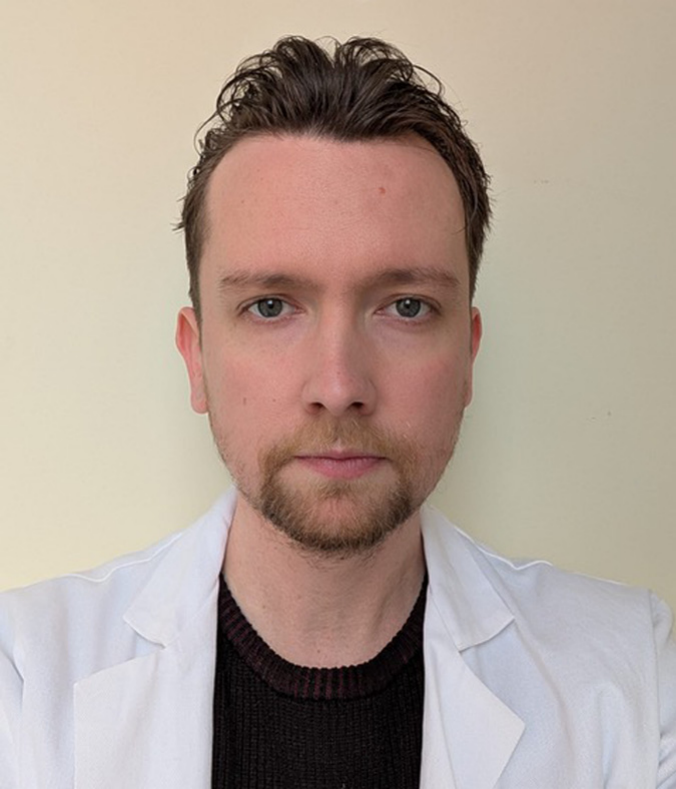


## MEET THE FIRST AUTHOR

***Meet the First Author(s)* is an editorial feature within *RNA*, in which the first author(s) of research-based papers in each issue have the opportunity to introduce themselves and their work to readers of *RNA* and the RNA research community. Alexandre D'Halluin is the first author of this paper, “A *Mycobacterium tuberculosi*s Mbox controls a conserved, small upstream ORF via a translational expression platform and Rho-dependent termination of transcription.” Alexandre is a postdoc interested in bacterial regulatory RNA and RNA degradation, from their identification to their characterization. This work was done in Kristine Arnvig's laboratory during his first postdoc, at University College London in the UK, where he was interested in the regulation of riboswitches and premature termination of transcription in *M. tuberculosis*. Since then, he has moved to Ciarán Condon's laboratory in France to focus on post-transcriptional regulation of sporulation in *B. subtilis*.**

**What are the major results described in your paper and how do they impact this branch of the field?**

In our previous study, we showed that most *M. tuberculosis* riboswitches were associated with Rho-dependent termination of transcription and translation of small peptides. In this work, we characterized one of them, the magnesium riboswitch, which operates through a translational platform coupled with premature Rho-dependent termination of transcription. To our knowledge, this dual mechanism of regulation is the first case reported for an Mbox in bacteria. The riboswitch regulates a new small peptide, which is highly expressed and conserved across Mycobacteria, but for which a function remains to be unveiled. Our results provide another example of an uncommon mechanism of regulation in *M. tuberculosis* compared to well-known model organisms.

**What led you to study RNA or this aspect of RNA science?**

The ability of bacteria to adapt quickly to their environment has always piqued my curiosity. Regulating RNA level and mRNA expression is one of the quickest ways for microorganisms to respond to a stress, either through the action of small RNA, premature termination of transcription, or RNA decay. While working on post-transcriptional regulation, my attention has also been focused on the role of cryptic small ORF encoded by regulatory RNA.

**During the course of these experiments, were there any surprising results or particular difficulties that altered your thinking and subsequent focus?**

At the beginning of the project, I was very surprised to identify a lot of unannotated small peptides within riboswitches; the Mbox uORF2 was one of the first I had mapped and validated in vivo. The Mbox is known to be regulated via a transcriptional platform and intrinsic terminator, which we first expected to occur with Rho-dependent termination of transcription in *M. tuberculosis* as intrinsic terminators are rare. The identification of the two small upstream ORFs was surprising at first sight, and very exciting as we suspect them to be linked directly with premature termination of transcription. Indeed, translational expression platforms highly dominate in *M. tuberculosis*, and the Mbox is not an exception.

**If you were able to give one piece of advice to your younger self, what would that be?**

After all these years, I would mainly insist on one piece of advice: it is essential to plan the research at different levels as the unexpected is likely to happen regularly: (i, short term) What experiments should be done by next week; (ii, mid-term) What objectives and questions should be answered in the next 6 months; and (iii, long term) What are the long-term objectives of this project? Those plans can help to focus on the essential, to establish priorities and get an idea of the time left to answer a maximum of questions from the project.

**What are your subsequent near- or long-term career plans?**

I have more recently worked on the role of post-transcriptional regulation in the pathway to sporulation in *B. subtilis* as a postdoctoral researcher. I will seek a position that helps me to lead projects in this field where regulatory RNA and RNases are known to play a major role while their characterization and function are still yet to be discovered.
